# Aqua­{*N*-[1-(2-oxidophen­yl)ethyl­idene]-l-serinato}copper(II) monohydrate

**DOI:** 10.1107/S1600536809045292

**Published:** 2009-11-04

**Authors:** Gan-Qing Zhao, Da-Ming Tian, Yong-Jun Han, Ling-Wei Xue, Qin-Long Peng

**Affiliations:** a School of Chemistry and Chemical Engineering, Pingdingshan University, Pingdingshan 467000, People’s Republic of China; b Department of Chemical Engineering, Henan University of Urban Construction, Pingdingshan 467044, People’s Republic of China

## Abstract

In the title compound, [Cu(C_11_H_11_NO_4_)(H_2_O)]·H_2_O, each Cu^II^ ion is four-coordinated by one N and two O atoms from the tridentate Schiff base ligand, and by one O atom from the coordinated water mol­ecule in a distorted square-planar geometry. Inter­molecular O—H⋯O hydrogen bonds link complex mol­ecules and solvent water mol­ecules into flattened columns propagated in [100].

## Related literature

For general background to the chemistry of transition metal complexes with Schiff base ligands composed of salicylaldehyde, 2-formyl­pyridine or their analogues, and α-amino acids, see: Casella & Guillotti (1983 ▶); Vigato & Tamburini (2004 ▶); Ganguly *et al.* (2008 ▶). For related structures, see: Usman *et al.* (2003 ▶); Parekh *et al.* (2006 ▶); Basu Baul *et al.* (2007 ▶). For details of the synthesis, see: Plesch *et al.* (1997 ▶).
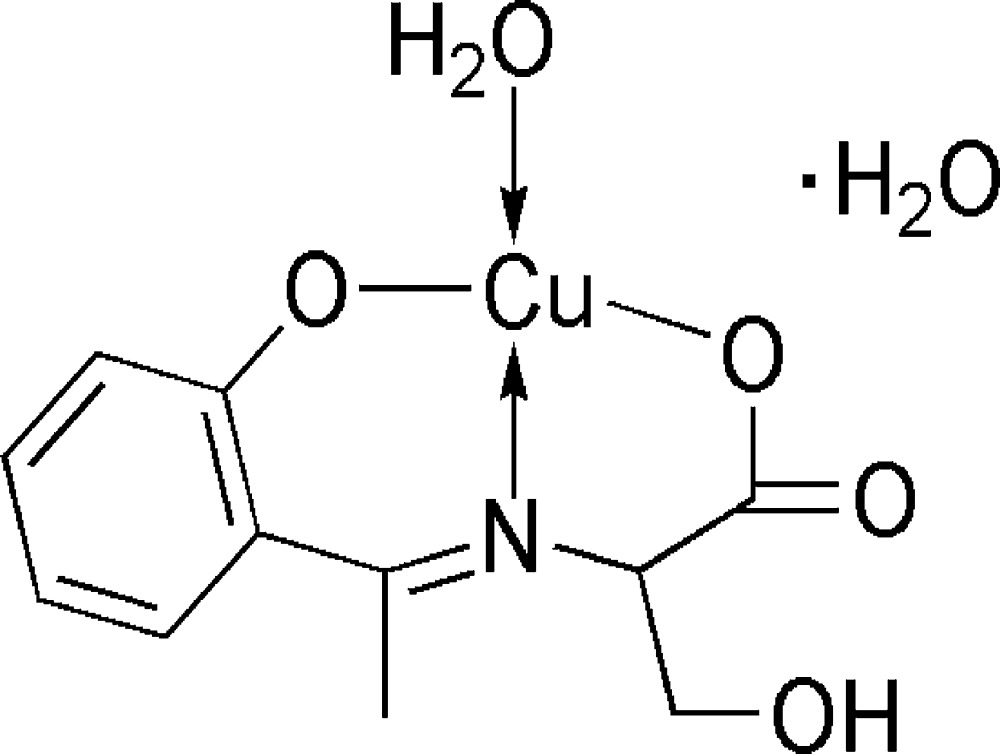


## Experimental

### 

#### Crystal data

[Cu(C_11_H_11_NO_4_)(H_2_O)]·H_2_O
*M* *_r_* = 320.78
Orthorhombic, 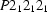

*a* = 5.6701 (9) Å
*b* = 13.788 (2) Å
*c* = 15.536 (2) Å
*V* = 1214.6 (3) Å^3^
*Z* = 4
Mo *K*α radiation
μ = 1.82 mm^−1^
*T* = 296 K
0.25 × 0.20 × 0.20 mm

#### Data collection

Bruker SMART APEXII CCD diffractometer
Absorption correction: multi-scan (*SADABS*; Sheldrick, 1996 ▶) *T*_min_ = 0.659, *T*_max_ = 0.712
6314 measured reflections
2149 independent reflections
2038 reflections with *I* > 2σ(*I*)
*R*_int_ = 0.027

#### Refinement

*R*[*F* ^2^ > 2σ(*F* ^2^)] = 0.022
*wR*(*F* ^2^) = 0.053
*S* = 1.09
2149 reflections
176 parameters
H-atom parameters constrained
Δρ_max_ = 0.21 e Å^−3^
Δρ_min_ = −0.24 e Å^−3^
Absolute structure: Flack (1983 ▶), 869 Friedel pairs
Flack parameter: 0.011 (13)

### 

Data collection: *APEX2* (Bruker, 2008 ▶); cell refinement: *SAINT* (Bruker, 2008 ▶); data reduction: *SAINT*; program(s) used to solve structure: *SHELXS97* (Sheldrick, 2008 ▶); program(s) used to refine structure: *SHELXL97* (Sheldrick, 2008 ▶); molecular graphics: *SHELXTL* (Sheldrick, 2008 ▶); software used to prepare material for publication: *SHELXTL*.

## Supplementary Material

Crystal structure: contains datablocks global, I. DOI: 10.1107/S1600536809045292/cv2643sup1.cif

Structure factors: contains datablocks I. DOI: 10.1107/S1600536809045292/cv2643Isup2.hkl

Additional supplementary materials:  crystallographic information; 3D view; checkCIF report

## Figures and Tables

**Table 1:** Hydrogen-bond geometry (Å, °)

*D*—H⋯*A*	*D*—H	H⋯*A*	*D*⋯*A*	*D*—H⋯*A*
O4—H4*A*⋯O3^i^	0.82	1.84	2.651 (3)	171
O1*W*—H1*WA*⋯O2*W* ^ii^	0.82	1.91	2.694 (3)	161
O1*W*—H1*WB*⋯O2^iii^	0.85	1.92	2.740 (3)	162
O2*W*—H2*WA*⋯O4	0.85	2.04	2.837 (3)	156
O2*W*—H2*WB*⋯O1^ii^	0.85	2.02	2.817 (3)	157

Symmetry codes: (i) 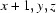; (ii) 
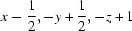
; (iii) 
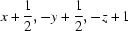
.

**Fig. 1.**
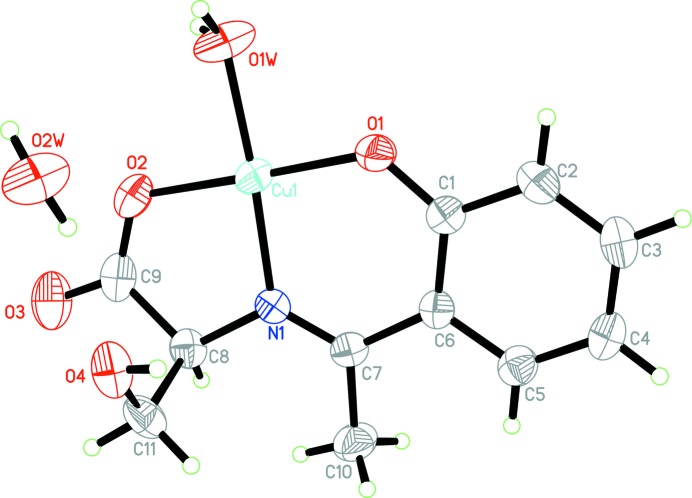
The structure of the title compound, showing 50% probability displacement ellipsoids and the atom-numbering scheme.

## supplementary crystallographic information

### Comment

In the past decades, significant progress has been achieved in understanding the chemistry of transition metal complexes with Schiff base ligands composed of salicylaldehyde, 2-formylpyridine or their analogues, and α-amino acids (Vigato & Tamburini, 2004; Ganguly *et al.*, 2008; Casella & Guillotti, 1983). A few stuctural studies have been performed on Schiff base complexes derived from 2-Hydroxyacetophenone and animo acids (Usman *et al.*, 2003; Basu Baul *et al.*, 2007; Parekh *et al.*, 2006). We report here the crystal structure of the title compound (I).

The asymmetric unit of (I) contains a monomeric square-planar coordinated Cu^II^ complex and one solvate water molecule (Fig. 1). The Cu—N bond length is 1.9335 (19) Å, while Cu—O bond lengths lie in the range 1.8595 (18)-1.9677 (18) Å.

The crystal structure is stabilized by O—H···O type hydrogen bonds (Table 1), which link complex molecules and solvent water molecules into flattened columns propagated in direction [100].

### Experimental

The title compound was synthesized as described in the literature (Plesch *et al.*, 1997). To L-serine (1.00 mmol) and potassium hydroxide (1.00 mmol) in 10 ml of methanol was added 2-Hydroxyacetophenone (1.00 mmol in 10 ml of methanol) dropwise. The yellow solution was stirred for 2.0 h at 333 K. The resultant mixture was added dropwise to copper (II) acetate monohydrate (1.00 mmol) in an aqueous methanolic solution (20 ml, 1:1 *v*/*v*), and heated with stirring for 2.0 h at 333 K. The dark green solution was filtered and left for several days, dark green crystals had formed that were filtered off, washed with water, and dried under vacuum.

### Refinement

All H atoms were positioned geometrically (C—H = 0.93-0.97 Å, O—H = 0.82-0.85 Å) and refined as riding, with *U*_iso_(H) = 1.2-1.5*U*_eq_ of the parent atom.

### Crystal data

**Table:**

[Cu(C_11_H_11_NO_4_)(H_2_O)]·H_2_O	*F*(000) = 660
*M**_r_* = 320.78	*D*_x_ = 1.754 Mg m^−^^3^
Orthorhombic, *P*2_1_2_1_2_1_	Mo *K*α radiation, λ = 0.71073 Å
Hall symbol: P 2ac 2ab	Cell parameters from 3823 reflections
*a* = 5.6701 (9) Å	θ = 2.6–27.3°
*b* = 13.788 (2) Å	µ = 1.82 mm^−^^1^
*c* = 15.536 (2) Å	*T* = 296 K
*V* = 1214.6 (3) Å^3^	Block, dark green
*Z* = 4	0.25 × 0.20 × 0.20 mm

### Data collection

**Table:**

Bruker SMART APEXII CCD diffractometer	2149 independent reflections
Radiation source: fine-focus sealed tube	2038 reflections with *I* > 2σ(*I*)
graphite	*R*_int_ = 0.027
φ and ω scans	θ_max_ = 25.0°, θ_min_ = 2.6°
Absorption correction: multi-scan (*SADABS*; Sheldrick, 1996)	*h* = −2→6
*T*_min_ = 0.659, *T*_max_ = 0.712	*k* = −16→16
6314 measured reflections	*l* = −18→18

### Refinement

**Table:**

Refinement on *F*^2^	Hydrogen site location: inferred from neighbouring sites
Least-squares matrix: full	H-atom parameters constrained
*R*[*F*^2^ > 2σ(*F*^2^)] = 0.022	*w* = 1/[σ^2^(*F*_o_^2^) + (0.0174*P*)^2^ + 0.2008*P*] where *P* = (*F*_o_^2^ + 2*F*_c_^2^)/3
*wR*(*F*^2^) = 0.053	(Δ/σ)_max_ = 0.001
*S* = 1.09	Δρ_max_ = 0.21 e Å^−^^3^
2149 reflections	Δρ_min_ = −0.24 e Å^−^^3^
176 parameters	Extinction correction: *SHELXL97* (Sheldrick, 2008), Fc^*^=kFc[1+0.001xFc^2^λ^3^/sin(2θ)]^-1/4^
0 restraints	Extinction coefficient: 0.0120 (11)
Primary atom site location: structure-invariant direct methods	Absolute structure: Flack (1983), 869 Friedel pairs
Secondary atom site location: difference Fourier map	Flack parameter: 0.011 (13)

### Special details

**Table:**

Geometry. All e.s.d.'s (except the e.s.d. in the dihedral angle between two l.s. planes) are estimated using the full covariance matrix. The cell e.s.d.'s are taken into account individually in the estimation of e.s.d.'s in distances, angles and torsion angles; correlations between e.s.d.'s in cell parameters are only used when they are defined by crystal symmetry. An approximate (isotropic) treatment of cell e.s.d.'s is used for estimating e.s.d.'s involving l.s. planes.
Refinement. Refinement of *F*^2^ against ALL reflections. The weighted *R*-factor *wR* and goodness of fit *S* are based on *F*^2^, conventional *R*-factors *R* are based on *F*, with *F* set to zero for negative *F*^2^. The threshold expression of *F*^2^ > σ(*F*^2^) is used only for calculating *R*-factors(gt) *etc*. and is not relevant to the choice of reflections for refinement. *R*-factors based on *F*^2^ are statistically about twice as large as those based on *F*, and *R*- factors based on ALL data will be even larger.

### Fractional atomic coordinates and isotropic or equivalent isotropic displacement parameters (Å^2^)

**Table:**

	*x*	*y*	*z*	*U*_iso_*/*U*_eq_	
Cu1	0.18718 (6)	0.09862 (2)	0.532935 (19)	0.02947 (11)	
C1	0.5839 (5)	−0.02296 (17)	0.50130 (16)	0.0286 (6)	
C2	0.7793 (5)	−0.04620 (19)	0.44924 (16)	0.0378 (7)	
H2	0.8084	−0.0092	0.4003	0.045*	
C3	0.9287 (5)	−0.12205 (18)	0.46860 (19)	0.0390 (6)	
H3	1.0554	−0.1360	0.4326	0.047*	
C4	0.8899 (5)	−0.17732 (19)	0.54164 (19)	0.0382 (7)	
H4	0.9903	−0.2284	0.5553	0.046*	
C5	0.7019 (6)	−0.15591 (18)	0.59347 (16)	0.0340 (6)	
H5	0.6780	−0.1933	0.6425	0.041*	
C6	0.5428 (5)	−0.07988 (16)	0.57613 (15)	0.0268 (6)	
C7	0.3496 (5)	−0.06175 (17)	0.63784 (15)	0.0283 (6)	
C8	0.0332 (5)	0.03559 (19)	0.69616 (16)	0.0304 (6)	
H8	−0.0484	−0.0238	0.7139	0.036*	
C9	−0.1447 (4)	0.10765 (19)	0.66029 (18)	0.0354 (6)	
C10	0.3232 (7)	−0.1327 (2)	0.71079 (18)	0.0469 (8)	
H10A	0.4539	−0.1260	0.7496	0.070*	
H10B	0.3200	−0.1976	0.6883	0.070*	
H10C	0.1788	−0.1199	0.7410	0.070*	
C11	0.1466 (5)	0.0833 (2)	0.77456 (16)	0.0393 (7)	
H11A	0.0281	0.0931	0.8187	0.047*	
H11B	0.2671	0.0408	0.7978	0.047*	
N1	0.2116 (4)	0.01209 (14)	0.63046 (12)	0.0247 (4)	
O1	0.4525 (3)	0.05113 (12)	0.47659 (11)	0.0372 (4)	
O2	−0.0953 (4)	0.14741 (13)	0.58792 (13)	0.0409 (5)	
O3	−0.3178 (4)	0.12658 (15)	0.70381 (15)	0.0552 (6)	
O4	0.2492 (3)	0.17369 (14)	0.75275 (13)	0.0412 (5)	
H4A	0.3869	0.1654	0.7383	0.062*	
O1W	0.1373 (4)	0.18950 (14)	0.43700 (13)	0.0523 (6)	
H1WA	−0.0026	0.1897	0.4236	0.078*	
H1WB	0.1923	0.2466	0.4318	0.078*	
O2W	0.2141 (4)	0.33399 (16)	0.63903 (14)	0.0550 (6)	
H2WA	0.2036	0.2776	0.6607	0.066*	
H2WB	0.1045	0.3569	0.6077	0.066*	

### Atomic displacement parameters (Å^2^)

**Table:**

	*U*^11^	*U*^22^	*U*^33^	*U*^12^	*U*^13^	*U*^23^
Cu1	0.02747 (17)	0.02884 (16)	0.03211 (16)	0.00077 (15)	−0.00322 (15)	0.00440 (13)
C1	0.0274 (14)	0.0282 (12)	0.0303 (12)	−0.0033 (11)	−0.0041 (12)	−0.0019 (11)
C2	0.0400 (17)	0.0408 (15)	0.0324 (14)	−0.0022 (13)	0.0062 (13)	−0.0010 (11)
C3	0.0339 (15)	0.0387 (15)	0.0446 (15)	0.0005 (12)	0.0076 (15)	−0.0103 (13)
C4	0.0327 (15)	0.0318 (13)	0.0500 (17)	0.0059 (12)	−0.0027 (14)	−0.0036 (14)
C5	0.0379 (15)	0.0273 (12)	0.0368 (14)	0.0001 (14)	−0.0022 (15)	−0.0017 (11)
C6	0.0254 (13)	0.0248 (13)	0.0302 (12)	−0.0027 (11)	−0.0013 (11)	−0.0041 (10)
C7	0.0267 (15)	0.0286 (12)	0.0296 (12)	−0.0029 (11)	−0.0028 (12)	0.0015 (10)
C8	0.0228 (14)	0.0329 (13)	0.0354 (14)	−0.0055 (12)	0.0067 (12)	0.0012 (11)
C9	0.0238 (15)	0.0309 (13)	0.0517 (16)	−0.0047 (13)	−0.0008 (13)	−0.0103 (14)
C10	0.0446 (19)	0.0500 (16)	0.0459 (16)	0.0087 (16)	0.0081 (17)	0.0212 (13)
C11	0.0340 (17)	0.0547 (17)	0.0291 (12)	−0.0025 (14)	0.0084 (12)	−0.0040 (13)
N1	0.0224 (11)	0.0263 (10)	0.0254 (10)	−0.0043 (10)	−0.0002 (10)	−0.0010 (8)
O1	0.0353 (10)	0.0426 (10)	0.0336 (10)	0.0061 (9)	0.0052 (9)	0.0097 (8)
O2	0.0347 (11)	0.0372 (10)	0.0509 (12)	0.0099 (9)	−0.0037 (10)	0.0023 (9)
O3	0.0287 (12)	0.0614 (14)	0.0754 (15)	0.0059 (11)	0.0118 (13)	−0.0107 (11)
O4	0.0291 (13)	0.0436 (10)	0.0508 (12)	−0.0034 (8)	0.0022 (9)	−0.0159 (9)
O1W	0.0461 (15)	0.0468 (12)	0.0639 (13)	−0.0085 (10)	−0.0166 (11)	0.0273 (11)
O2W	0.0374 (12)	0.0585 (13)	0.0690 (14)	−0.0080 (12)	−0.0080 (12)	0.0245 (11)

### Geometric parameters (Å, °)

**Table:**

Cu1—O1	1.8595 (18)	C8—N1	1.473 (3)
Cu1—N1	1.9335 (19)	C8—C9	1.522 (4)
Cu1—O2	1.936 (2)	C8—C11	1.527 (4)
Cu1—O1W	1.9677 (18)	C8—H8	0.9800
C1—O1	1.322 (3)	C9—O3	1.220 (3)
C1—C2	1.408 (4)	C9—O2	1.282 (3)
C1—C6	1.422 (3)	C10—H10A	0.9600
C2—C3	1.379 (4)	C10—H10B	0.9600
C2—H2	0.9300	C10—H10C	0.9600
C3—C4	1.385 (4)	C11—O4	1.416 (3)
C3—H3	0.9300	C11—H11A	0.9700
C4—C5	1.368 (4)	C11—H11B	0.9700
C4—H4	0.9300	O4—H4A	0.8200
C5—C6	1.409 (4)	O1W—H1WA	0.8200
C5—H5	0.9300	O1W—H1WB	0.8502
C6—C7	1.477 (3)	O2W—H2WA	0.8500
C7—N1	1.289 (3)	O2W—H2WB	0.8500
C7—C10	1.505 (3)		
			
O1—Cu1—N1	95.37 (8)	C9—C8—C11	106.9 (2)
O1—Cu1—O2	177.99 (9)	N1—C8—H8	109.6
N1—Cu1—O2	85.87 (9)	C9—C8—H8	109.6
O1—Cu1—O1W	89.08 (9)	C11—C8—H8	109.6
N1—Cu1—O1W	175.46 (9)	O3—C9—O2	124.8 (3)
O2—Cu1—O1W	89.66 (9)	O3—C9—C8	118.0 (3)
O1—C1—C2	116.9 (2)	O2—C9—C8	117.1 (2)
O1—C1—C6	124.9 (2)	C7—C10—H10A	109.5
C2—C1—C6	118.2 (2)	C7—C10—H10B	109.5
C3—C2—C1	122.0 (2)	H10A—C10—H10B	109.5
C3—C2—H2	119.0	C7—C10—H10C	109.5
C1—C2—H2	119.0	H10A—C10—H10C	109.5
C2—C3—C4	119.9 (3)	H10B—C10—H10C	109.5
C2—C3—H3	120.1	O4—C11—C8	111.2 (2)
C4—C3—H3	120.1	O4—C11—H11A	109.4
C5—C4—C3	119.2 (2)	C8—C11—H11A	109.4
C5—C4—H4	120.4	O4—C11—H11B	109.4
C3—C4—H4	120.4	C8—C11—H11B	109.4
C4—C5—C6	123.1 (2)	H11A—C11—H11B	108.0
C4—C5—H5	118.4	C7—N1—C8	121.9 (2)
C6—C5—H5	118.4	C7—N1—Cu1	126.91 (17)
C5—C6—C1	117.5 (2)	C8—N1—Cu1	110.98 (15)
C5—C6—C7	118.5 (2)	C1—O1—Cu1	126.28 (16)
C1—C6—C7	124.0 (2)	C9—O2—Cu1	114.80 (17)
N1—C7—C6	121.7 (2)	C11—O4—H4A	109.5
N1—C7—C10	121.3 (2)	Cu1—O1W—H1WA	109.5
C6—C7—C10	116.9 (2)	Cu1—O1W—H1WB	127.5
N1—C8—C9	110.2 (2)	H1WA—O1W—H1WB	109.1
N1—C8—C11	111.0 (2)	H2WA—O2W—H2WB	121.1

### Hydrogen-bond geometry (Å, °)

**Table:**

*D*—H···*A*	*D*—H	H···*A*	*D*···*A*	*D*—H···*A*
O4—H4A···O3^i^	0.82	1.84	2.651 (3)	171
O1W—H1WA···O2W^ii^	0.82	1.91	2.694 (3)	161
O1W—H1WB···O2^iii^	0.85	1.92	2.740 (3)	162
O2W—H2WA···O4	0.85	2.04	2.837 (3)	156
O2W—H2WB···O1^ii^	0.85	2.02	2.817 (3)	157

Symmetry codes: (i) *x*+1, *y*, *z*; (ii) *x*−1/2, −*y*+1/2, −*z*+1; (iii) *x*+1/2, −*y*+1/2, −*z*+1.
